# Characterization and trypanocidal activity of a β-lapachone-containing drug carrier

**DOI:** 10.1371/journal.pone.0246811

**Published:** 2021-03-04

**Authors:** Juliana M. C. Barbosa, Caroline D. Nicoletti, Patrícia B. da Silva, Tatiana G. Melo, Débora O. Futuro, Vitor F. Ferreira, Kelly Salomão

**Affiliations:** 1 Laboratório de Biologia Celular Instituto Oswaldo Cruz, Fundação Oswaldo Cruz, Rio de Janeiro, Rio de Janeiro, Brazil; 2 Laboratório de Síntese Orgânica Aplicada, Instituto de Química, Universidade Federal Fluminense, Niterói, RJ, Brazil; 3 Faculdade de Farmácia, Universidade Federal Fluminense, Niterói, Rio de Janeiro, Brazil; 4 Laboratório de Ultraestrutura Celular, Instituto Oswaldo Cruz, Fundação Oswaldo Cruz, Rio de Janeiro, Rio de Janeiro, Brazil; Cleveland State University, UNITED STATES

## Abstract

The treatment of Chagas disease (CD), a neglected parasitic condition caused by *Trypanosoma cruzi*, is still based on only two drugs, nifurtimox (**Nif**) and benznidazole (**Bz**), both of which have limited efficacy in the late chronic phase and induce severe side effects. This scenario justifies the continuous search for alternative drugs, and in this context, the natural naphthoquinone β-lapachone (**β-Lap**) and its derivatives have demonstrated important trypanocidal activities. Unfortunately, the decrease in trypanocidal activity in the blood, high toxicity to mammalian cells and low water solubility of **β-Lap** limit its systemic administration and, consequently, clinical applications. For this reason, carriers as drug delivery systems can strategically maximize the therapeutic effects of this drug, overcoming the above mentioned restrictions. Accordingly, the aim of this study is to investigate the *in vitro* anti-*T*. *cruzi* effects of **β-Lap** encapsulated in2-hydroxypropyl-β-cyclodextrin (**2HP-β-CD**) and its potential toxicity to mammalian cells.

## Introduction

Chagas disease (CD), caused by the flagellate protozoan *Trypanosoma cruzi*, is an endemic illness that affects 21 countries in Latin America. Currently, it is considered by the World Health Organization (WHO) one of the twenty neglected tropical diseases, affecting more than 5 million people worldwide **[1–3]**. Even 111 years after its discovery, the etiological treatment of CD is still restricted to two nitroheterocycles: benznidazole (**Bz**) and nifurtimox (**Nif**) **[4]**. Their effectiveness varies with the phase of the infection, the dose and period of treatment, and the age and geographical origin of the patient **[5]**. Additionally, severe adverse reactions and limited efficacy in the chronic phase justify the urgent need for new drugs for CD treatment **[6]**. For this reason, there is an intensive research effort focused on the search for alternative natural and synthetic new drugs to be used alone or in combination with Bz or with other repurposed drugs **[7, 8]**.

Quinones are present in nature and play an important role in energy production in microorganisms, plants and animals **[9]**. Naphthoquinones are privileged structures in medicinal chemistry due to their structural and biological characteristics, especially against tumor cells and pathogenic protozoa **[10, 11]**. The fundamental feature of quinones is their ease of reduction and, therefore, their ability to act as oxidizing or dehydrogenating agents. Their cytotoxicity has been associated with redox cycling, DNA fragmentation, inhibition of human DNA topoisomerase I and II, bioreductive alkylation via the generation of quinonemethides, arylation of the thiol groups of proteins and free radical generation **[12–15]**. β-Lapachone (**β-Lap**), a naphthoquinone isolated from the heartwood of trees of the Bignoniaceae family (Tabebuiasp.), has several bioactive effects, with anti-inflammatory, hepatoprotective, anticancer and antimicrobial properties **[16–22]**. The anti-*T*. *cruzi* activity of **β-Lap** has been intensively studied by the Docampo group since the 1980s **[23, 24]**. Unfortunately, the trypanocidal effects of **β-Lap** are abrogated in the presence of blood and serum, suggesting that it could be inactivated either by reduction in the presence of oxyhemoglobin or by interaction with serum proteins **[25]**. In addition, the low water solubility of **β-Lap** limits its systemic administration and clinical use **[26]**, requiring the search for delivery systems **[27, 28]**. Another approach is the formulation of drugs using colloidal systems, such as liposomes and nanoparticles, to (a) specifically target the affected tissues, (b) improve drug bioavailability and (c) reduce the required dose, thus decreasing toxicity **[27, 29, 30]**. In this context, cyclodextrins (**CDs**), a family of cyclic oligosaccharides consisting of a macrocyclic ring of glucose subunits joined by α-1,4 glycosidic bonds, are extensively used in the pharmaceutical industry to obtain nanocomplexes of different drugs. This class of guest molecules can improve drug solubility, stability, and bioavailability **[31–33]**. Complexation strategies have been successfully explored in studies with **Bz** to increase its plasma concentration and reduce its *in vitro* cytotoxicity without impairing biological activity **[30, 34–37]**.

The aim of the present work is to determine the *in vitro* trypanocidal activity of a new nanostructured **CD** formulation loaded with **β-Lap** and its potential toxicity to mammalian cells.

## Materials and methods

### Chemicals

Analytical grade solvents were used. Reagents and 2-hydroxypropyl-β-cyclodextrin (**2HP-β-CD**) were purchased from Sigma-Aldrich or Acros Chemical Co. Ltd., and β-Lapachone was synthesized following a previously reported procedure **[38]** (**Fig 1**). The reaction was monitored by thin-layer chromatography carried out using 0.25 mm Merck silica gel plates (60F-254) with UV light as the visualizing agent. The crude product was purified via silica gel (Merck 70–230 mesh) column chromatography using a gradient mixture of hexane and ethyl acetate. Yields refer to purified compounds obtained by chromatographic techniques and confirmed by characterization data obtained from melting points.

**Fig 1:**
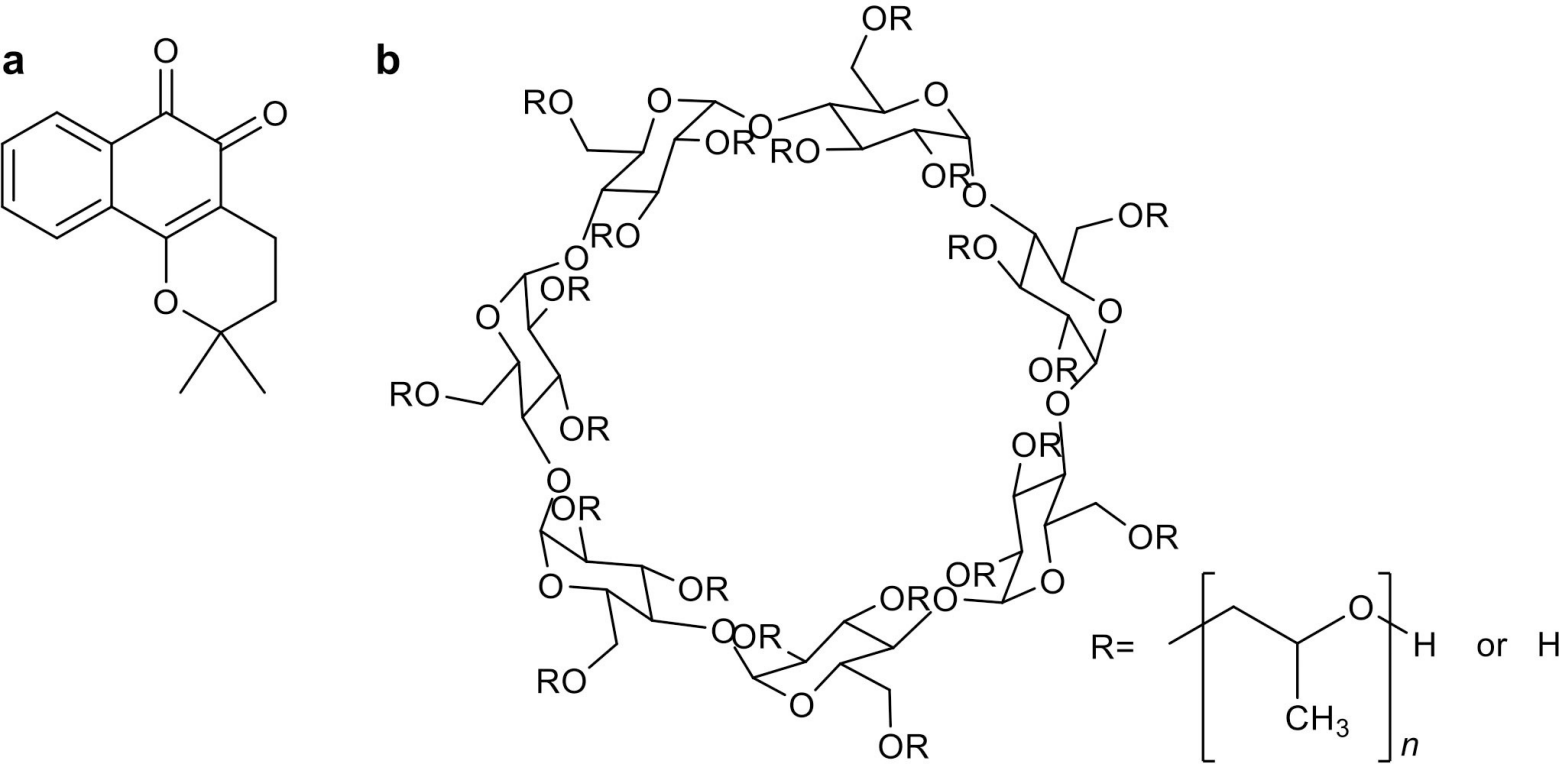
Chemical structure of (a) β-Lap and (b) 2HP-β-CD.

### Preparation of the β-Lap:CD complex

The inclusion complex (**β-Lap:CD**) of β-LAP and 2-HP-β-CD was prepared by complete dissolution of 2-HP-β-CD (500 mg) in phosphate-buffered saline, pH 7.4 (1 mL) and adding **β-LAP** (20 mg) with magnetic stirring for 72 h followed by freeze-drying **[28]**.

For all experiments, stock solutions of **β-Lap:CD** and **β-Lap** were prepared in PBS and dimethyl sulfoxide, respectively. The final concentration of the solvent in the experiments never exceeded 0.6%, a concentration that is known to not exert toxicity to the parasite or host cells.

### Biological activity

All experiments dealing with animals were performed in accordance with Brazilian Law 11.794/2008 and regulations of the National Council of Animal Experimentation Control under license L038/2018 from the Ethics Committee for Animal Use of the Oswaldo Cruz Institute (CEUA/IOC). Male mice between 3–4 weeks were housed at a maximum of 5 individuals per cage, kept in a specific-pathogen-free (SPF) room at 20 to 22°C under a 12/12 h light/dark cycle at 50 to 60% humidity and provided sterilized water and chow *ad libitum* **[39]**. The animals will be euthanized to obtain the parasites on the eighth day after infection to carry out the experiments. The procedure will be carried out by injecting intraperitoneally, in the lower right half of the abdomen, a sedative-analgesic drug associated with anesthetic overdose. After application of the lethal dose of anesthesia we will wait for the loss of pain reflexes (analyzed by clamping the paw) and will be performed cervical dislocation to ensure the animal’s death. Animals infected with *T*. *cruzi* that will be used to obtain the parasites will be observed daily for signs that indicate intense suffering. Some of the parameters to be observed are: stay in the corner of the box with the local snout down, prostration and difficulty in eating, weight loss greater than 20% of body weight and goose bumps. When two or more factors change, denoting suffering, or animal will be euthanized.

### *In vitro* activity of β-Lap:CD and β-Lap against the Tulahuen strain of *T*. *cruzi*

Culture-derived trypomastigotes of the Tulahuen strain (DTU IV) expressing the *Escherichia coli* β-galactosidasegene **[40]** were used to infect L929 mouse fibroblasts (MOI 10:1). After 2 h of interaction, the cultures were washed and maintained for 48 h to establish infection, and then **β-Lap:CD** or **β-Lap** was added and incubated for 96 h at 37°C. After this period, 50 μL of chlorophenol red glycoside (500 μM) (CPRG, Sigma-Aldrich) was added to 0.5% Nonidet P40 solution (Sigma-Aldrich), and the plates were incubated for another 18 h. Next, the absorbance was measured at 570 nm, and the results were expressed as the percent inhibition of infection or *T*. *cruzi* growth inhibition. The standard drug **Bz** was used as a control **[41]**.

### *In vitro* activity of β-Lap:CD and β-Lap against the Y strain of *T*. *cruzi*

Evaluation of the **β-Lap:CD** and **β-Lap** activities against intracellular amastigote forms (Y strain) was performed using primary cultures of mouse embryo heart muscle cells (HMCs) as host cells. Briefly, hearts of 18-day-old mouse embryos were fragmented and dissociated with trypsin and collagenase in PBS. Thereafter, isolated cells were cultivated in Dulbecco’s modified Eagle medium (DMEM) containing 7% fetal bovine serum (FBS; Cultilab, São Paulo, Brazil), 2.5 mM CaCl2, 1 mM L-glutamine (Sigma), 2% chicken embryo extract and 1% penicillin/streptomycin solution (Life Technologies, São Paulo, Brazil) and then plated onto gelatin-coated glass coverslips maintained at 37°C in a 5% CO_2_ atmosphere **[42]**. HMCs were infected with bloodstream trypomastigotes (MOI 10:1), and after 24 h, the cultures were washed to remove the noninternalized parasites. Next, **β-Lap:CD** or **β-Lap** was added (2 to 0.7 μM) for 24 h at 37°C. The cultures were fixed and stained with Diff-Quick (Laborclin) and examined by light microscopy to determine the percent infected cells and infection index (II), which corresponds to the number of parasites/100 cells **[43]**. The results are also expressed using the IC_50_/24 h, which corresponds to the concentration that produces a 50% decrease in the II **[39]**.

Bloodstream trypomastigotes (Y strain; DTU II) were obtained from the blood of infected Swiss-Webster mice at the peak of parasitemia by heart puncture and isolated by differential centrifugation and resuspended in DME supplemented with 10% fetal calf serum medium **[44]**. The parasites (5×10^6^ cells/mL) were incubated with **β-Lap:CD** or **β-Lap** for 24 h in two experimental conditions: at 37°C in the absence of blood or at 4°C in the presence of 5% benznidazole, the standard drug, which was used as positive control **[11, 43]**.

Epimastigote forms (Y strain) were maintained axenically at 28°C with weekly transfers in LIT medium and were harvested during the exponential phase of growth. The parasites were incubated with **β-Lap:CD** or **β-Lap** for 24 h at 28°C. For both trypomastigotes and epimastigotes, cell counts were performed in a Neubauer chamber, and the activity was expressed as the IC_50_/24 h, corresponding to the concentration that led to 50% lysis of the parasites **[11]**.

### *In vitro* toxicity of β-Lap:CD and β-Lap to mammalian cells

Cytotoxicity assays were performed using two models, the mouse fibroblast cell line L929, obtained from the American Type Culture Collection (Manassas, VA) (using RPMI-1640 medium, pH 7.2 plus 10% foetal bovine serum and 2 mM L-glutamine) and HMCs using DMEMS. In both cases, 5 × 10^4^ cells in 200 μL of the appropriate culture medium were added to each well of a 96-well microtiter plate and incubated with **β-Lap:CD** or **β-Lap** for 24 h or 96 h at 37°C. Afterwards, PrestoBlue (Invitrogen) was added at a 1:10 ratio in medium, and the microplates were incubated for 5 h. The absorbance was measured at 570 and 600 nm using a spectrophotometer, as recommended by the manufacturer. The results are expressed as the difference in the percent reduction between treated and untreated cells, with the LC_50_ being the concentration that leads to damage of 50% of the mammalian cells. The selectivity index (SI) was calculated by the ratio between the LC_50_ and IC_50_, the latter of which was the concentration that led to 50% lysis/proliferation inhibition of the parasites **[45]**.

### Transmission electron microscopy analysis

Epimastigotes (Y strain, 5 × 10^6^ cells/mL) were treated for 24 h with **β-Lap:CD** or **β-Lap** at the concentrations corresponding to their IC_50_/24 h values. The parasites were fixed with 2.5% glutaraldehyde in 0.1 M Na-cacodylate buffer (pH 7.2) for 40 min at 25°C and post fixed for 20 min at 25°C with 1% OsO_4_, 0.8% potassium ferricyanide and 2.5 mM CaCl_2_ in buffer. The samples were dehydrated in an ascending acetone series and embedded in Polybed 812 resin. Ultrathin sections were stained with uranyl acetate and lead citrate and examined with a JEOL JEM1011 transmission electron microscope (Tokyo, Japan) (Technological Platform of Electronic Microscopy at the Institute of Oswaldo Cruz) **[39]**.

### Statistical analysis

Data are expressed as the arithmetic means ± SD of at least three independent experiments. All statistical tests were performed using the Mann-Whitney t test and ANOVA in IBM SPSS Statistics 22.0 software (IBM Corporation, Armonk, New York, USA), p value ≤ 0.05 was considered significant.

## Results

First, following our well-established flow chart flux **[41]**, the activity against the intracellular forms of the Tulahuen strain (DTU IV) was performed using the L929-infected cell line. Infected cultures were treated with **β-Lap:CD** or **β-Lap** (0.15–10 μM) for 96 h and analyzed by colorimetric analysis. As presented in **Table 1**, encapsulation increased trypanocidal activity (**β-Lap:CD** IC_50_/96h = 0.60 ± 0.05 μM) compared to free naphthoquinone (**β-Lap** IC_50_/96h = 2.21 ± 1.25 μM). Although the toxicity of **β-Lap:CD** to L929 was approximately two times higher than that of **β-Lap**, leading to SI values of 6.6 and 3.5, respectively, the formulation presented higher selectivity.

**Table 1:** Effects of β-Lap:CD and β-Lap on the intracellular forms of *T*. *cruzi* (Tulahuen strain) and toxicity to L929 fibroblasts.

	IC_50_/96 h(μM)	LC_50_/96 h (μM)	SI
**β-Lap:CD**	0.60 ± 0.05^a^	3.94 ± 0.02	6.6^b^
**β-Lap**	2.21 ± 1.25	7.76 ± 0.02	3.5
**Bz**	2.16 ± 0.98	190.6 ± 13.4^c^	88.2

^a^Mean ± SD of at least three independent experiments;

^b^ selectivity index (SI) = LC_50_/IC_50_;

^c^Simões-Silva et al. 2017 **[46]**.

To expand the analysis to other *T*. *cruzi* strains and mammalian host cells, **β-Lap:CD** and **β-Lap** were screened against intracellular forms of the Y strain (DTU II) using HMCs as host cells. To determine the concentrations to be used in the trypanocidal assay, first, the toxicity to the host cell was determined. Since the LC_50_/24 h values for HMCs were 10.88 ± 4.19 and 6.03 ± 1.40 μM for **β-Lap:CD** and **β-Lap**, respectively, the range of nontoxic concentrations for subsequent assays were established as 0.7 to 2 μM.

**Fig 2:**
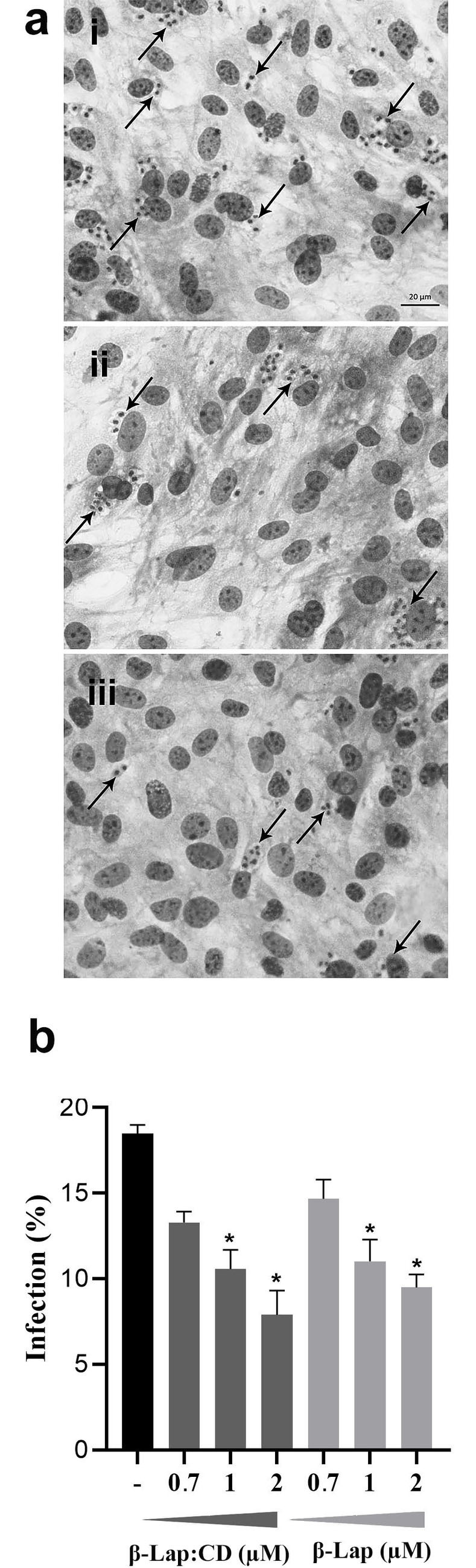
Effects of β-Lap:CD and β-Lap on *T*. *cruzi* (Y strain)-infected HMCs after 24 h of treatment: (a) HMCs were incubated with trypomastigotes (MOI 10:1) for 24 h. The cultures were washed and then β-Lap:CD or β-Lap was added (0.7 to 2 μM) for another 24 h at 37°C. Then, the cultures were fixed and stained with Diff-Quick, and the infection index was determined in [i] untreated cultures, [ii] β-Lap:CD (IC_50_/24h = 1.59 ± 0.40 μM) and [iii] β-Lap (IC_50_/24h = 1.24 ± 0.49 μM). Thick black arrows correspond to intracellular forms of the parasite (Bar = 20 μM). (b) Graphic representation of the dose-dependent inhibition of the percent of infected cells. *Asterisks represent the significant difference in relation to the untreated control group (p ≤ 0.05).

Light microscopy illustrated the inhibition of HMC infection by the Y strain of *T*. *cruzi* after a 24 h of treatment with 1 μM **β-Lap:CD** or **β-Lap** (**Fig 2A**). Quantification of the infected and treated cultures showed dose-dependent inhibition of the infection by both **β-Lap:CD** and **β-Lap** (**Fig 2B**); a similar range of inhibition (20–60%) was observed at concentrations between 0.7 and 2 μM (**Fig 2B**).

In sequence, the activities of **β-Lap:CD** and **β-Lap** on bloodstream trypomastigotes of the Y strain were evaluated. Two experimental conditions were used: in DMES in the absence and presence of blood. Without blood, no difference in trypanocidal activity between **β-Lap:CD** (IC_50_/24h = 5.85 ± 0.57 μM) and **β-Lap** (IC_50_/24h = 6.13 ± 0.65 μM) was observed (**Table 2**). The presence of 5% blood and a reduction of the temperature to 4°C led to a decrease in trypanocidal activity in both cases; however, this decrease was 2-fold higher for **β-Lap:CD**.

**Table 2:** Effects of β-Lap:CD and β-Lap on the trypomastigote forms of *T*. *cruzi* (Y strain) under two experimental conditions.

Compound	IC_50_ (μM)	
	0 % blood	5 % blood
**β-Lap:CD**	6.13 ± 0.65^a^	404.98 ± 24.70
**β-Lap**	5.85 ± 0.57	198.85 ± 18.55
**Bz**	8.81 ± 1.08	103.60 ± 0.60^b^
**CD**	>1000	

^a^ Mean ± SD of at least three independent experiments;

^b^ Da Silva Jr et al. 2008 **[47]**.

**Fig 3:**
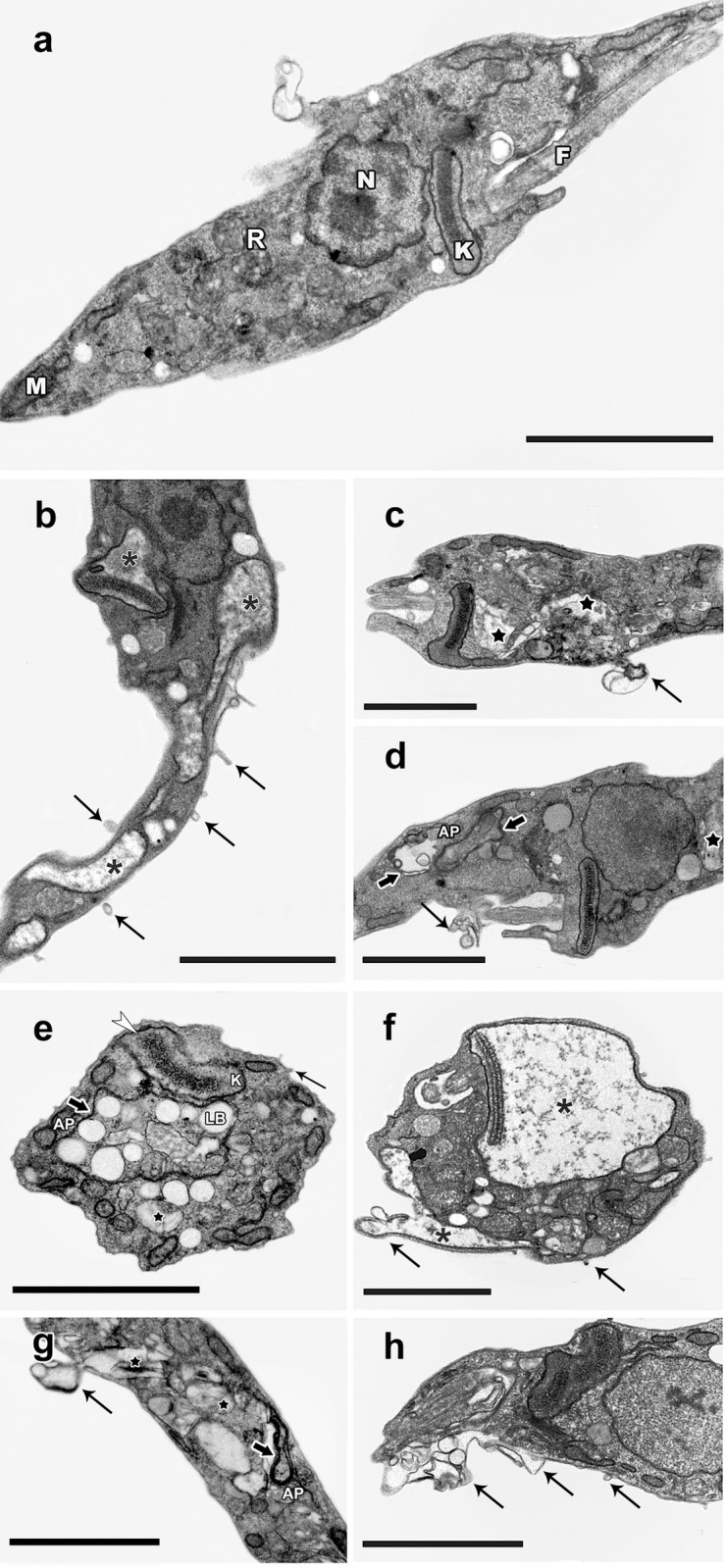
Ultrastructural alterations in *T*. *cruzi* epimastigotes treated with 10 μMβ-Lap:CD or β-Lap. (a) Untreated parasites showing the typical elongated body, with normal morphology of the mitochondrion (M), nucleus (N), flagellum (F), reservosomes (R) and kinetoplast (K). (b-d) β-Lap:CD; (e-h). β-Lap:CD and β-Lap generated similar phenotypic changes, with swelling of the mitochondria (asterisks), disorganization of reservosomes (black star) and blebs in the plasma membrane and flagellum (thick black arrows), presence of large kinetoplasts (K) with an altered kDNA compacting pattern (white arrowhead), concentric membrane structures (black arrows) involving lipid bodies (LB) and reservosomes with characteristics of autophagosomes (AP) and the occasional formation of inner vesicles. Bars: 2 μm.

In order to investigate whether the encapsulation of **β-Lap** alters the way in which this naphthoquinone acts on the parasite ultrastructure, epimastigotes of *T*. *cruzi* (Y strain) were treated with **β-Lap:CD** and **β-Lap** at concentrations corresponding to their IC_50_/24 h values of 11.7 ± 1.6 and 10.2 ± 2.2 μM, respectively. After incubation for 24 h, the materials were processed for transmission electron microscopy and analyzed. In both cases, the most frequent alterations were mitochondrial swelling, disorganization of reservosomes and blebbing in the plasma and flagellum membranes. Epimastigotes treated with **β-Lap:CD** showed some other alterations, such as large kinetoplasts with altered kDNA compacting patterns, concentric membrane structures involving lipid bodies and reservosomes with the characteristic morphology of autophagosome annal formation of inner vesicles (**Fig 3**).

## Discussion

The focus of the present study was to assess the *in vitro* trypanocidal activity of a new nanostructured CD formulation loaded with **β-Lap**. In recent years, this quinone has attracted considerable attention, particularly in cancer research **[48–50]**. The trypanocidal activity of **β-Lap** and its derivatives has also been extensively demonstrated *in vitro* and *in vivo* **[51, 52]**. However, low water solubility **[26]** and inactivation of **β-Lap** in the presence of blood and serum limit its systemic administration and clinical applications for the treatment of CD **[13, 25]**. It has been hypothesized that encapsulation of this drug could solve these problems, since the use of cyclodextrin in nanocomplexes with **Bz** was well received, leading to improved solubility and decreased *in vitro* cytotoxicity **[30, 34]**.

Recently, our group described for the first time the inclusion of **β-Lap** in cyclodextrin and its activity against *T*. *cruzi*, observing that complexation enhanced the SI compared to the free form of this drug **[37]**. In the present work, aiming to deepen the study of **β-Lap** complexes, free and encapsulated forms of the quinone were investigated on the three evolutionary forms of the parasite with emphasis on the forms relevant to mammalian infection (amastigotes and trypomastigotes) **[2]**. Additionally, to expand this study, two different strains of *T*. *cruzi* were employed: Tulahuen (DTU VI), considered susceptible to nitro derivatives, and Y (DTU II), partially resistant to Bz **[53, 54]**.

The encapsulation of **β-Lap** improved significantly the activity of the quinone against intracellular amastigotes of the Tulahuen strain in L929 fibroblasts by 3.7 times (p = 0.028); however, the difference in the SI value was less noted because the complex was more toxic to the mouse fibroblast lineage than the quinone itself. On the other hand, for the Y strain, no significant differences were observed in the activity of **β-Lap:CD** over **β-Lap** against amastigotes interiorized in cardiac cells, showing that the encapsulation did not interfere with the trypanocidal activity of the quinone. The distinct behavior or the Tulahuen and Y strains could be due to differences in the standardized protocols employed, and we also cannot discard the differences in susceptibility between both strains.

The encapsulation of **β-Lap** did not interfere with the quinone activity against bloodstream trypomastigotes (Y strain) in our standard conditions **[43]**, i.e., DMES medium at 37°C, with IC_50_ values of 5.85 ± 0.57 and 6.13 ± 0.65 μM for **β-Lap:CD** and **β-Lap**, respectively. Blood addition (5%) and temperature reduction (from 37°C to 4°C) led to a substantial decrease in trypanocidal activity, as expected; however, this decrease was 34-fold higher for the cyclodextrin complex (66X) than for β-Lap. In view of these results, it is hypothesized that the cyclodextrin formulation could interact with cholesterol and other blood lipids **[55–57]** and form an insoluble complex **[58]** that is responsible for the decrease in activity of the encapsulated form of **β-Lap**.

Ultrastructural analysis of the treated epimastigotes indicates that the mechanism of action of **β-Lap** is preserved with encapsulation, since the phenotypic changes observed with **β-Lap:CD** treatment are similar to those induced by **β-Lap**, as shown in the present work and in the literature **[59, 60]**. In this context, studies on **β-Lap** encapsulation should be continued, aiming to reverse problems related to the bioavailability of this drug. The results obtained with the Tulahuen strain encourage the development of nanostructures loaded with **β-Lap,** since the interaction of new formulations with cholesterol could improve drug stability, reduce the binding to plasma proteins and avoid precipitation **[61–63]**.

## Supporting information

S1 Graphical abstract (JPG)
