# Supplementary figures and images for: Characterization and trypanocidal activity of a β-lapachone-containing drug carrier
Source: PLoS One. 2021 Mar 4;16(3):e0246811. doi: 10.1371/journal.pone.0246811 (PMC7932091; doi:10.1371/journal.pone.0246811)

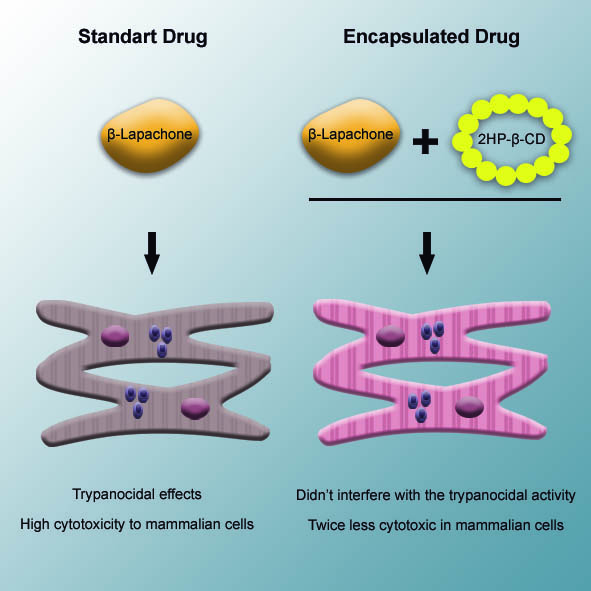

Supplement: S1 Graphical abstract — (JPG) [file pone.0246811.s001.jpg]
